# Using Optogenetic Dyadic Animal Models to Elucidate the Neural Basis for Human Parent–Infant Social Knowledge Transmission

**DOI:** 10.3389/fncir.2021.731691

**Published:** 2021-09-29

**Authors:** Victoria Leong, Gao Xiang Ham, George J. Augustine

**Affiliations:** ^1^Psychology, School of Social Sciences, Nanyang Technological University, Singapore, Singapore; ^2^Department of Psychology, University of Cambridge, Cambridge, United Kingdom; ^3^Lee Kong Chian School of Medicine, Nanyang Technological University, Singapore, Singapore

**Keywords:** social learning, optogenetics, dyadic EEG, parent–infant, neural synchrony

## Introduction: Social Learning Across Species

Healthy early development depends on a warm reciprocal relationship between parent and offspring, where parent and infant interact in close temporal co-ordination as if engaged in a “dyadic dance” of glances, gestures, smiles and words (Stern, 1985; Gianino and Tronick, 1988). Most, if not all, early learning takes place during these well-choreographed social exchanges, which support cultural knowledge transmission from parent to offspring using verbal and non-verbal forms of communication and behavioural modelling. Such vicarious knowledge transmission through social interaction (rather than direct experience) is known as social learning (Bandura, 1971; Csibra and Gergely, 2009). Tomasello (2014) argues that human mastery of these “second-personal social relations” (Darwall, 2006)—in which social partners share and create *joint* knowledge, intentionality and goals—has accelerated the rise of the human species through “cultural intelligence” (Herrmann et al., 2007).

One important and early developing form of social learning is social referencing. Here, a social partner's actions and emotions are used to form one's own understanding of a situation and guide behaviour (Feinman, 1982). Two main forms of social referencing are commonly recognised. *Instrumental* social referencing—also termed observational learning—refers to the use of others' actions to shape behaviour (cf. Bandura's Bobo doll experiment; Bandura, 1992), as occurs during imitation. *Affective* social referencing refers to the use of others' emotional expressions for event appraisal (Campos, 1983; Hornik and Gunnar, 1998). Affective social referencing - the focus of this article - is well-studied in human infants (Feinman, 1982; Hornik and Gunnar, 1998; Clement and Dukes, 2016), and develops over the first year of life. By 10–12 months of age, infants begin to seek information from others in novel situations and use this information to regulate their own affect and behaviour (Feinman et al., 1992). For example, human infants at this age will avoid crossing a short visual cliff (Sorce et al., 1985), show less interaction with toys (Gunnar and Stone, 1984; Hornik et al., 1987) and be less friendly to strangers when their mothers show negative emotion toward these objects or individuals as compared to neutral or happy emotional expressions (Feinman and Lewis, 1983; Feinman et al., 1986). Such social knowledge transmission from parent to offspring is therefore crucial during early life in helping infants to safely explore and learn about their physical and social environments.

An analogous rudimentary form of social learning occurs in animal species such as mice, an example of which is the social transmission of food preferences (STFP). When a naive mouse interacts with a social partner who has eaten a novel flavoured food, this social interaction confers familiarity with the flavour, and the naive mouse will now eat more of the familiarised food than completely new food (Galef, 2003; Wrenn, 2004). Crucially, as in human subjects, the learning of food preferences occurs through face-to-face social interaction: when the naive mouse sniffs the breath, face and whiskers of the demonstrator mouse. During murine development, this form of social learning underpins intergenerational transmission of food choices between adult mice and pups, allowing weanlings who are exploring their food options to learn vicariously about safe foods (and avoid eating poisoned foods) that their elders have experienced (Silverman et al., 2010). Therefore, in both human and murine species, knowledge transmission through social interaction with adult caregivers plays a vital role in shaping the developing youngling's understanding of the world and how to interact successfully with it. However, much still remains unknown about the neural mechanisms and processes that support this form of vicarious social learning.

## Neural Synchrony: An Evolutionarily Conserved Mechanism For Social Knowledge Transmission?

**Figure 1:**
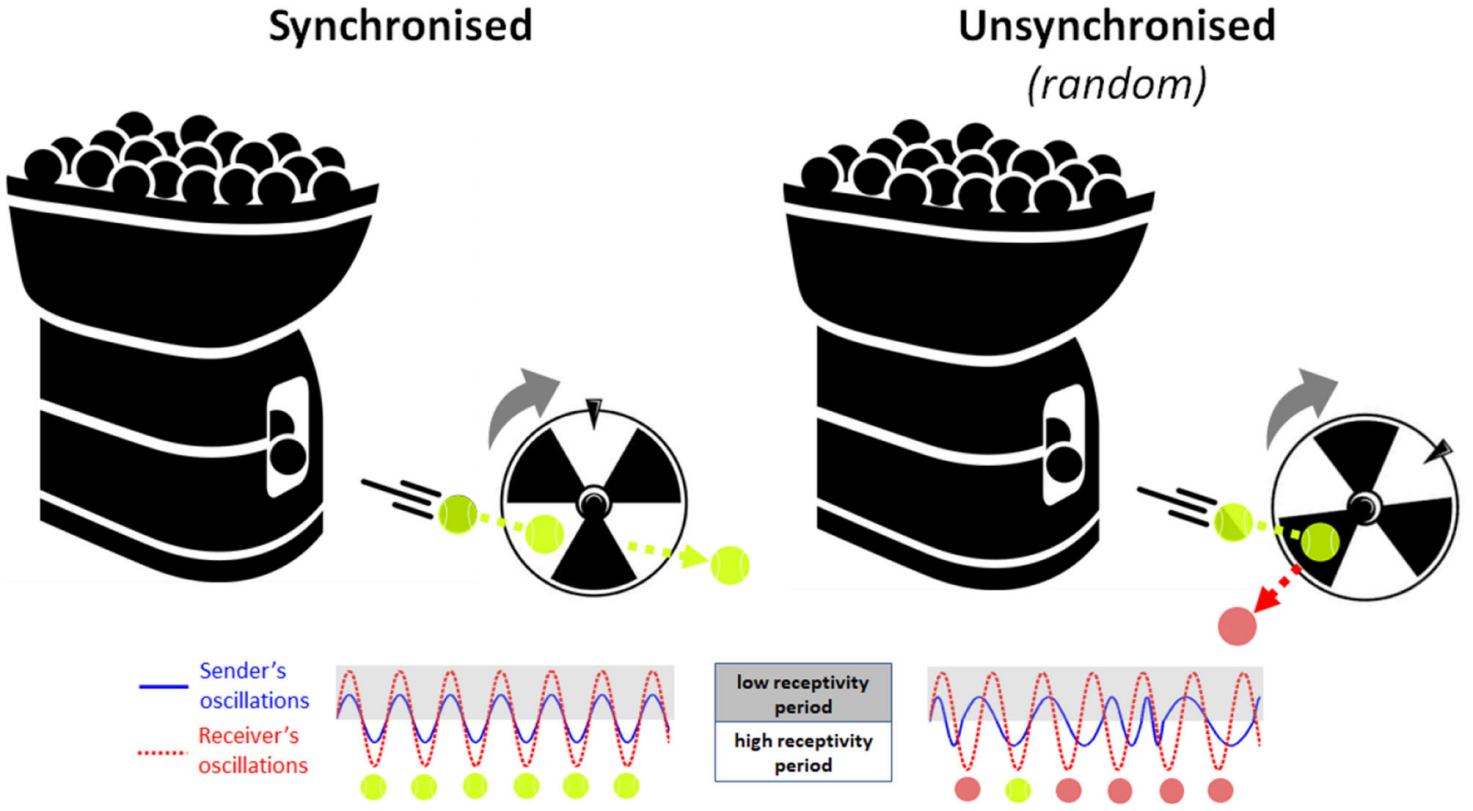
Analogical illustration of the Learning through Interpersonal Neural Coupling (LINC) hypothesis. Here, the tennis ball machine (information sender) attempts to fire balls through the blades of a rotating wheel (information receiver). (Left) If the timing of ball firing is well-synchronised to the rotation pattern of the wheel, then every ball passes successfully between the blades every time. (Right) Conversely, if the balls are fired randomly without reference to the phase of the turning wheel, then many balls will hit the blades and bounce out and only a few will pass through by chance. Therefore, synchronisation permits all the information (balls) to be safely transmitted from the sender (machine) to the receiver (wheel), where the timing of ball firing and wheel rotation represent the neuronal oscillation patterns of the information sender (blue solid line) and the information receiver (red dotted line), respectively.

Neuronal oscillations are observed across many species and support basic processes in information encoding, memory and attention. In humans, perception relies on neural oscillatory processes in the cortex that shape our conscious experience (Buzsaki, 2006). Research suggests that the oscillatory phase of neural activity at the time a stimulus occurs may relate to the excitability of cortical neuron populations and to the magnitude of event-related responses elicited by the stimulus (Lakatos et al., 2008; Busch et al., 2009; although see Ruzzoli et al., 2019). Accordingly, perceptual stimuli that are delivered during a high-excitability phase of neural oscillations are more likely to be detected and encoded than stimuli that arrive at a low-excitability, inhibitory phase (Busch et al., 2009; Mathewson et al., 2009). Extending this conceptual framework to the social (dyadic) domain, the phase of on-going neural oscillations in the child's brain may similarly determine the efficacy of capturing information from their social partner “in the moment.” However, because social interaction is an active process, rather than a passive one, this presents the possibility that social partners may actively modulate each other's neural state, using salient social cues (like gaze or touch) to transiently reset the phase of their partner's neural oscillations. For example, a parent may initiate eye contact to reset the phase of her child's neural oscillations to match her own oscillations, triggering a short-term increase in parent-child neural synchrony. During this brief state of high interpersonal synchrony, parents' and infants' neural receptivity periods are mutually well-aligned in time or “coupled.” This allows pieces of information delivered by the parent (e.g., spoken words) to be presented at optimal times for encoding (learning) by the infant “receiver.” As a simple analogy, imagine a scenario where a tennis ball machine fires balls repetitively through a rotating turbine wheel (see Figure 1). If the timing of ball firing is well-synchronised to the rotation pattern of the wheel, then every ball passes successfully between the blades every time. Conversely, if the balls are fired randomly without reference to the phase of the turning wheel, then many balls will hit the blades and bounce out and only a few will pass through by chance. Therefore, synchronisation is the key to transmitting all the information (balls) from the sender (machine) safely through to the receiver (wheel), where the timing of ball firing and wheel rotation represent the neuronal oscillation patterns of the sender and receiver, respectively. This two-brain synchronisation model of social learning, or *Learning through Interpersonal Neural Coupling (LINC) Hypothesis* predicts that social learning is “gated” by interpersonal neural synchronisation, and that transient states of synchronisation are achieved through the use of social signals that reset the phase of on-going oscillations.

In human adult-infant dyads, recent dyadic-electroencephalography (EEG) studies have shown that during social interaction, adult-infant neural oscillation patterns can indeed become transiently synchronised (Leong et al., 2017; Santamaria et al., 2020; Wass et al., 2020). Consistent with the LINC Hypothesis, it has recently been found that stronger neural synchronisation between human mothers and their infants (as measured by an index of phase-locking) does indeed predict a higher likelihood of successful affective social referencing by infants (Leong et al., 2019). Further, natural increases in interpersonal phase synchronisation are associated with the use of social teaching signals such as eye contact and prosodically enhanced maternal speech (Leong et al., 2017, 2019), which suggests that such social signals may indeed increase interpersonal synchronisation through mechanisms such as oscillatory phase-resetting. However, the non-invasive constraints of human infant studies prevent a deeper interrogation and understanding of the exact neural structures and circuits that generate interpersonal synchrony. Further, the correlational nature of human infant studies does not permit causal inference of whether neural synchrony is *necessary* for social learning or merely a meta-phenomenon of the process—a long-standing debate in the field of two-person neuroscience.

## A Precision Tool For Studying Social Learning Neural Mechanisms: The Dyadic Optogenetic Mouse Model

Although interpersonal neural synchrony was documented first in humans, this mechanism may in fact be evolutionarily conserved to subserve social interaction behaviour across human and non-human animal species. Recent animal research suggests that interbrain neural synchrony predicts a diverse set of social interaction behaviours in rodents (Kingsbury et al., 2019) and in bats (Zhang and Yartsev, 2019). For example, Kingsbury et al. (2019) performed microendoscopic calcium imaging between pairs of freely interacting mice and found that neural activity in the dorsomedial prefrontal cortex (dmPFC) was highly correlated between mice during social interaction. This strong adult-adult dyadic neural correlation was dependent on features of the ongoing social interaction rather than on shared sensory input from a common environment or concurrent behaviour. Further, dmPFC correlation between mice predicted their future social interaction patterns and dominance relationship. In a similar study with bats, Zhang and Yartsev (2019) used wireless electrophysiology to perform simultaneous recordings of neural local field potentials (LFPs) and spiking activity in pairs of spontaneously interacting bats. Both LFP power and spike activity were highly correlated between bats over multiple timescales, ranging from seconds to hours. Further, the degree of neural correlation covaried with the extent of social interaction between bats, spiking just before interactions were initiated. These initial animal studies indicate that socially induced synchronisation of neural activity between conspecifics may be a fundamental mechanism that drives and shapes social interaction patterns and preferences. Here, we specifically propose that interpersonal neural synchronisation supports social knowledge transmission across species.

If interpersonal neural synchronisation is in fact causally necessary for social learning, then targeted manipulation of neural synchronicity within the dyad should also influence the success of social learning. Optogenetic methods provide an optimal way to test this causal link. Optogenetics is a revolutionary technology that permits genetically defined, light-based control of neural circuits, providing unparalleled spatial, temporal and genetic resolution for the study of neural and cognitive mechanisms in living organisms (Boyden et al., 2005; Kim et al., 2017). In this approach, transgenic animals (e.g., rats or mice) express light-gated ion channels, pumps or receptors [i.e., opsins such as channelrhodopsin-2 (ChR2) and halorhodopsin] in specific types of neurons. The activity of these neurons can be selectively increased (photostimulation) or decreased (photoinhibition) by exposure to light of the appropriate wavelength, allowing experimental control of neural activity—and the cognitive functions that these neural circuits subserve—at the flick of a light switch. Animal models have long been used to study basic learning and social behaviour, but in recent years, optogenetic technology has increasingly been employed to study complex social behaviour in animals (e.g., anxiety, depression and aggression; Yizhar, 2012). In regard to the study of social learning, optogenetics may be employed to assess the success of transmission of food preference from a mouse dam to her pup (i.e., STFP) during either synchronous or asynchronous stimulation of parent and infant brain regions. Recently, Yang et al. (2021) demonstrated the feasibility of a dyadic optogenetic approach in pairs of freely interacting adult mice, through the use of implantable, miniaturised wireless stimulation devices. This is an important methodological advance as it permits precise control and experimental manipulation of interpersonal synchrony *at the neural source*, allowing the direct tests of causality on observed social behaviour that will significantly advance understanding in our field.

## Future Research Avenues and Challenges

If dyadic optogenetic technology can be successfully implemented in infant mice, parent-pup mouse optogenetic models could revolutionise the study of early social learning and be used to elucidate the precise neural pathways and mechanisms by which responsive caregiving and parenting behaviour act to scaffold early neurodevelopment and cognitive skills in offspring. These models can also be extended to study the aetiology of social developmental disorders such as autism, ADHD and other learning disabilities, as well as disorders of parent–child interaction and bonding, which occur during maternal depression and other forms of early life stress.

However, a dyadic optogenetic mouse model involving pups has never been created before, which presents new and significant technical challenges. For example, although the expression of channelrhodopsin and other optogenetic probes can be robust by age P21, this may impose a lower limit on the age at which pups may be tested and (depending on the exact promoter employed) may preclude the study of very early perinatal behaviour. Also, although lightweight head-mounted wireless devices (e.g., weighing as little as 20 mg) are now available (Montgomery et al., 2015), this still presents a significant load for very small pups and may impose restrictions on movement, feeding and other social interactive behaviour that would be of interest.

A second major challenge pertains to the design and selection of animal social experimental paradigms that are suitable for use with very young animals, and also closely parallel social behaviour in human infants, to permit meaningful comparison of cross-species data. For example, here we suggest that the social transmission of food preference in mice is a form of social learning that is analogous to social referencing by human infants. Although learning occurs through social interaction in both cases, the modality of information and its transfer (and therefore the sensorimotor pathways involved) are different. In the mouse paradigm, the information transmitted and learned is primarily olfactory (although auditory cues such as ultra-short-range high-frequency vocalisations may also be involved in shaping such social interactive behaviour; Warren et al., 2020) whereas human infants rely more on visual and auditory information from the caregiver's facial and vocal expressions, gestures and actions when performing social referencing (Sorce et al., 1985; Leong et al., 2019). This difference in perceptual processing pathways may be non-trivial when seeking to draw inferences from animal to human learning behaviour. It would be even more complex—and perhaps impossible—to draw extrapolations to higher social mental functions, such as theory of mind and other social mentalising abilities (e.g., inferring others' intentions, goals, beliefs), possibly limiting the utility of animal models in advancing understanding in these more complex areas of social cognition.

Nonetheless, the advent of dyadic optogenetic technology is a boon and could—within the next decade—fundamentally remake the landscape of developmental social neuroscience and neuropsychiatry.

## Author Contributions

All authors listed have made a substantial, direct and intellectual contribution to the work, and approved it for publication.

## Funding

This research is supported by the Ministry of Education, Singapore, under its Academic Research Fund Tier 1 [RG99/20 to VL and GA; RG152/18 (NS) to VL].

## Conflict of Interest

The authors declare that the research was conducted in the absence of any commercial or financial relationships that could be construed as a potential conflict of interest.

## Publisher's Note

All claims expressed in this article are solely those of the authors and do not necessarily represent those of their affiliated organizations, or those of the publisher, the editors and the reviewers. Any product that may be evaluated in this article, or claim that may be made by its manufacturer, is not guaranteed or endorsed by the publisher.

## References

[B1] Bandura A. (1971). Social Learning Theory. New York: General Learning Press.

[B2] Bandura A. (1992). Social cognitive theory of social referencing in Social Referencing and the Social Construction of Reality in Infancy, eds. S. Feinman (Boston, MA: Springer). 10.1007/978-1-4899-2462-9_8

[B3] Boyden E. S. Zhang F. Bamberg E. Nagel G. Deisseroth K. (2005). Millisecond-timescale, genetically targeted optical control of neural activity. Nat. Neurosci. 8, 1263–1268. 10.1038/nn1525 16116447

[B4] Busch N. A. Dubois J. VanRullen R. (2009). The phase of ongoing EEG oscillations predicts visual perception. J. Neurosci. 29, 7869–7876. 10.1523/JNEUROSCI.0113-09.2009 19535598 PMC6665641

[B5] Buzsaki G. (2006). Rhythms of the Brain. Oxford: Oxford University Press.

[B6] Campos J. J. (1983). The importance of affective communication in social referencing: a commentary on Feinman. Merrill Palmer Q. 29, 83–87.

[B7] Clement F. Dukes D. (2016). Social appraisal and social referencing: two components of affective social learning. Emot. Rev. 2016, 1–9. 10.1177/1754073916661634

[B8] Csibra G. Gergely G. (2009). Natural pedagogy. Trends Cogn. Sci. 13, 148–153. 10.1016/j.tics.2009.01.005 19285912

[B9] Darwall S. (2006). The Second-Person Standpoint: Respect, Morality, and Accountability. Cambridge, MA: Harvard University Press

[B10] Feinman S. (1982). Social referencing in infancy. Merrill Palmer Q. 28, 445–470.

[B11] Feinman S. Lewis M. (1983). Social referencing at ten months: a second-order effect on infants' responses to strangers. Child Dev. 54, 878–887. 10.2307/1129892 6617309

[B12] Feinman S. Roberts D. Hsieh K.-F. Sawyer D. Swanson D. (1992). A critical review of social referencing in infancy, in Social Referencing and the Social Construction of Reality in Infancy, eds. S. Feinman (New York, NY: Plenum)

[B13] Feinman S. Roberts D. Morissette P. L. (1986). The effect of social referencing on 12-month-olds' responses to a stranger's attempts to make friends. Paper presented at the Fifth International Conference on Infant Studies, Los Angeles.

[B14] Galef B. G. Jr, and Whiskin, E. E. (2003). Socially transmitted food preferences can be used to study long-term memory in rats. Learn. Behav. 31, 160–164. 10.3758/BF03195978 12882374

[B15] Gianino A. Tronick E. Z. (1988). The mutual regulation model: the infant's self and interactive regulation and coping and defensive capacities, in Stress and Coping Across Development, eds. T. M. Field, P. M. McCabe, and N. Schneiderman (Hillsdale, NJ, US: Lawrence Erlbaum Associates, Inc), 47–68.

[B16] Gunnar M. R. Stone C. (1984). The effects of positive maternal affect on infant responses to pleasant, ambiguous, and fear-provoking toys. Child Dev. 55, 1231–1236. 10.2307/1129992

[B17] Herrmann E. Call J. Hernandez-Lloreda M. V. Hare B. Tomasello M. (2007). Humans have evolved specialized skills of social cognition: the cultural intelligence hypothesis. Science 317, 1360–1366. 10.1126/science.1146282 17823346

[B18] Hornik R. Gunnar M. R. (1998). A descriptive analysis of infant social referencing. Child Dev. 59, 626–634. 10.2307/1130562 3383671

[B19] Hornik R. Risenhoover N. Gunnar M. (1987). The effects of maternal positive, neutral, and negative affective communications on infant responses to new toys. Child Dev. 58, 937–944. 10.2307/1130534

[B20] Kim C. K. Adhikari A. Deisseroth K. (2017). Integration of optogenetics with complementary methodologies in systems neuroscience. Nat. Rev. Neurosci. 18, 222–235. 10.1038/nrn.2017.15 28303019 PMC5708544

[B21] Kingsbury L. Huang S. Wang J. Gu K. Golshani P. Wu Y. E. Hong W. (2019). Correlated neural activity and encoding of behavior across brains of socially interacting animals. Cell 178, 429–446.e16. 10.1016/j.cell.2019.05.022 31230711 PMC6625832

[B22] Lakatos P. Karmos G. Mehta A. D. Ulbert I. Schroeder C. E. (2008). Entrainment of neuronal oscillations as a mechanism of attentional selection. Science 320, 110–113. 10.1126/science.1154735 18388295

[B23] Leong V. Byrne E. Clackson K. Harte N. Lam S. Wass S. (2017). Speaker gaze changes information coupling between infant and adult brains. Proc. Natl. Acad. Sci. USA (PNAS) 114, 13290–13295. 10.1073/pnas.1702493114 29183980 PMC5740679

[B24] Leong V. Noreika V. Clackson K. Georgieva S. Brightman L. Nutbrown R.. (2019). Mother-infant interpersonal neural connectivity predicts infants' social learning. PsyArXiv. 10.31234/osf.io/gueaq

[B25] Mathewson K. E. Gratton G. Fabiani M. Beck D. M. Ro T. (2009). To see or not to see: prestimulus α phase predicts visual awareness. J. Neurosci. 29, 2725–2732. 10.1523/JNEUROSCI.3963-08.2009 19261866 PMC2724892

[B26] Montgomery K. L. Yeh A. J. Ho J. S. Tsao V. Mohan Iyer S. Grosenick L.. (2015). Wirelessly powered, fully internal optogenetics for brain, spinal and peripheral circuits in mice. Nat. Methods 12, 969–974. 10.1038/nmeth.3536 26280330 PMC5507210

[B27] Ruzzoli M. Torralba M. Fernández L. M. Soto-Faraco S. (2019). The relevance of alpha phase in human perception. Cortex 120, 249–268. 10.1016/j.cortex.2019.05.012 31352236

[B28] Santamaria L. Noreika V. Georgieva S. Clackson K. Wass S. Leong V. (2020). Emotional valence modulates the topology of the parent-infant inter-brain network. NeuroImage 207:116341. 10.1016/j.neuroimage.2019.116341 31712166

[B29] Silverman J. L. Yang M. Lord C. Crawley J. N. (2010). Behavioural phenotyping assays for mouse models of autism. Nat. Rev. Neurosci. 11, 490–502. 10.1038/nrn2851 20559336 PMC3087436

[B30] Sorce J. F. Emde R. N. Campos J. Klinnert M. D. (1985). Maternal emotional signaling: Its effect on the visual cliff behavior of 1-year-olds. Dev. Psychol. 21, 195–200. 10.1037/0012-1649.21.1.195

[B31] Stern D. N. (1985). The Interpersonal World of the Infant. New York: Basic Books.

[B32] Tomasello M. A. (2014). A Natural History of Human Thinking. Harvard: Harvard University Press. 10.4159/9780674726369

[B33] Warren M. R. Clein R. S. Spurrier M. S. Roth E. D. Neunuebel J. P. (2020). Ultrashort-range, high-frequency communication by female mice shapes social interactions. Sci. Rep. 10:2637. 10.1038/s41598-020-59418-0 32060312 PMC7021676

[B34] Wass S. V. Whitehorn M. Marriot Haresign I. Phillips E. Leong V. (2020). Interpersonal neural synchrony and responsivity during early learning interactions. Trends Cogn. Sci. 24, 329–342. 10.1016/j.tics.2020.01.006 32160569

[B35] Wrenn C. C. (2004). Social transmission of food preference in mice. Curr. Protoc. Neurosci. 28, 5–8. 10.1002/0471142301.ns0805gs28 18428610

[B36] Yang Y. Wu M. Vázquez-Guardado A. Wegener A. J. Grajales-Reyes J. G. Deng Y.. (2021). Wireless Multilateral Devices for Optogenetic Studies of Individual and Social Behaviors. Berlin: Nature Publishing Group 10.1038/s41593-021-00849-x PMC8694284 33972800

[B37] Yizhar O. (2012). Optogenetic insights into social behavior function. Biol. Psychiatry. 71, 1075–1080. 10.1016/j.biopsych.2011.12.029 22341368

[B38] Zhang W. Yartsev M. M. (2019). Correlated neural activity across the brains of socially interacting bats. Cell 178, 413–428.e22. 10.1016/j.cell.2019.05.023 31230710 PMC6625887

